# Biocontrol Potentials of Antimicrobial Peptide Producing *Bacillus* Species: Multifaceted Antagonists for the Management of Stem Rot of Carnation Caused by *Sclerotinia sclerotiorum*

**DOI:** 10.3389/fmicb.2017.00446

**Published:** 2017-03-24

**Authors:** S. Vinodkumar, S. Nakkeeran, P. Renukadevi, V. G. Malathi

**Affiliations:** Department of Plant Pathology, Centre for Plant Protection, Tamil Nadu Agricultural UniversityCoimbatore, India

**Keywords:** *Bacillus*, *Sclerotinia*, carnation, anti-microbial peptide genes, antifungal activity, plant growth promotion

## Abstract

*Bacillus* species are widely exploited as biocontrol agents because of their efficiency in impeding various plant pathogens with multifaceted approach. In this study, *Bacillus* species were isolated from rhizosphere of various plants viz., carnations, cotton, turmeric, and bananas in Tamil Nadu state of India. Their potential to control the mycelial growth of *Sclerotinia sclerotiorum* was assessed *in vitro* by dual plate and partition plate techniques. *B. amyloliquefaciens* strain VB7 was much effective in inhibiting mycelial growth (45% inhibition of over control) and sclerotial production (100%). PCR detection of AMP genes revealed that *B. amyloliquefaciens* (VB7) had a maximum of 10 diverse antibiotic biosynthesis genes, namely, *ituD, ipa14, bacA, bacD, bamC, sfP, spaC, spaS, alba*, and *albF*, that resulted in production of the antibiotics iturin, bacilysin, bacillomycin, surfactin, subtilin, and subtilosin. Further, metabolites from *B. amyloliquefaciens* strains VB2 and VB7, associated with inhibition of *S. sclerotiorum*, were identified as phenols and fatty acids by gas chromatography mass spectrometry (GC-MS). Delivery of bacterial suspension of the effective strains of *Bacillus* spp. as root dip was found promising for the management of stem rot of cultivated carnations. Minimal percent disease incidence (4.6%) and maximum plant growth promotion was observed in the plants treated with *B. amyloliquefaciens* (VB7).

## Introduction

Globally, floriculture is a profitable business. Carnation (*Dianthus caryophyllus* L.) is important cut flower with greater stipulation in the global market because of its variety of colors. Major states involved in floriculture business in India are Tamil Nadu (55,000 ha area), Karnataka (30,600 ha area) and West Bengal (24,900 ha). Total cut flower production in India during 2013–2014 is 5,48,000 MT. Total production of carnations allover India is 6,590 MT. Assam ranks first in carnation production in India with 1,320 MT (Indian Horticulture Database, 2014). However, stem rot caused by *Sclerotinia sclerotiorum* (Lib.) de Bary is a devastating disease that causes major impacts on the cutflower industry. In India, stem rot of carnations, was first reported by Vinod Kumar et al. (2015a). *Sclerotinia* is a polyphagous fungi with wide host range, comprising 148 plant species (Saharan and Mehta, 2008). It is a robust pathogen with greater sustainability under adverse environmental conditions and survives as sclerotial bodies up to 8 years in soil (Adams and Ayers, 1979). Hundred per cent yield losses have been reported in susceptible crop plants (Purdy, 1979). *S. sclerotiorum*, infecting various crops, has been reported to exhibit resistance against fungicides. In brinjal and rapeseed, *Sclerotinia* showing resistance against fungicides like mancozeb, propineb, and carbendazim have been reported (Iqbal et al., 2003; Wang et al., 2014). Additionally, loss of non-targeted beneficial microflora and microfauna due to chemicals, have alerted scientists to the need for an alternate method of management. *Bacillus* species contain as many as 24 diverse AMP genes that allow the biosynthesis of antibiotics like iturin, bacilysin, bacillomycin, fengycin, surfactin, mersacidin, ericin, subtilin, subtilosin, and mycosubtilin (Chung et al., 2008). Antibiotics produced by the bacteria have specific modes of actions. Apart from this, *Bacillus* is known to produce volatile and non-volatile antimicrobial compounds that synergistically aid in curtailing plant diseases (Fernando et al., 2005; Mora et al., 2011). Antagonistic potential of *Bacillus* spp., against *S. sclerotiorum* has been explored in various host plants like mustard (Rahman et al., 2016), canola (Fernando et al., 2007; Kamal et al., 2015), soybean (Zhang and Xue, 2010), tomato (Abdeljalil et al., 2016). However, in case of carnations this is the first study regarding the management under protected cultivation. In this context, *Bacillus* species were explored as an effective and sustainable approach for the management of carnation stem rot under protected cultivation. Objectives of the present study includes (i) isolation and characterization of *Bacillus* species from rhizosphere, (ii) Identification of effective antagonist, (iii) Profiling the presence of diverse AMP genes, (iv) Evaluating the efficiency of Bacillus species under protected cultivation.

## Materials and methods

### Isolation and identification of *S. sclerotiorum*

The pathogen was isolated from an infected stem of carnation plant by following standard procedure (Tutte, 1969). Then the plates were incubated at 20 ± 2°C. The pathogen was identified through morphological and molecular characterization by sequencing 18S–28S rRNA genes (Vinod Kumar et al., 2015b).

### Testing the efficacy of *Bacillus* spp. against *Sclerotinia sclerotiorum in vitro*

Fifty strains of *Bacillus* spp., isolated from rhizosphere of various plants, viz., carnations, cotton, turmeric, and bananas and five of the most effective anti-fungal strains with known AMP gene profiles were obtained from the Culture Collection Centre (Department of Plant Pathology, Tamil Nadu Agricultural University, Coimbatore, India) for this study. Antifungal activity of the *Bacillus* spp. were evaluated through a dual culture technique (Xiaoning et al., 2014). Plates were incubated at 20 ± 2°C. After 7 days of incubation, percent inhibition (PI) of mycelial growth was calculated with the formula proposed by Dennis and Webster (1971). Mycelial plugs of the pathogen from the zone of interaction with antagonists were excised from the dual culture plates and subcultured on fresh PDA medium to assess the viability of antagonized mycelia. They were also observed under an optical microscope (Labomed—LX 400 V) and images were captured (IVU 5100).

### Characterization of 16S rRNA gene and AMP genes in *Bacillus* spp.

Ten strains showing the greatest antagonistic effects were selected among the 55 strains and were subjected for molecular characterization. Genomic DNA was extracted (Knapp and Chandlee, 1996) and 16S rRNA gene was characterized with BCF1 (5′CGGGAGGCAGCAGTAGGGAAT-3′) and BCR2 (5′-CTCCCCAGGCGGAGTGCTTT-3′) primer pair as proposed by Cano et al. (1994). Further, they were also screened for the presence of various AMP genes with their respective primer pairs (Table S1). A phylogenetic analysis was performed with MEGA 7 (Kumar et al., 2016) for the 16S rRNA gene of the study strains. The phylogeny was tested through neighbor joining method by 1,000 bootstrap replications and condensed with a cut-off value of 70%.

### *In vitro* antifungal activity of volatile compounds produced by *Bacillus* spp.

The ten most effective strains of *Bacillus* spp., as determined by the results of the dual plate assay, were used in this study. The efficacy of volatile compounds produced by *Bacillus* spp. were assessed using the partition plate technique (Fernando et al., 2005). *Bacillus* spp., were challenged with *S. sclerotiorum* on partition plates which enables the movement of volatiles alone without any direct contact between the microbes. The pathogen inoculated alone into the partition plate was maintained as control and incubated at 20 ± 2°C for 7 days. The experiment was replicated thrice. After 7 days of incubation, the percent inhibition of sclerotial production was calculated as proposed by Dennis and Webster (1971).

### Extraction and bioassay of crude metabolites of *Bacillus* spp.

Crude metabolites of the two most effective strains among the 55 dually challenged strains were extracted with ethyl acetate (Dheepa et al., 2016). The resulting condensate (100 mg) was dissolved in 1 ml of HPLC-grade methanol. The protective efficacy of the extract was assessed by the paper disc diffusion technique with 25, 50, and 75 μl of crude extracts (Hafidh et al., 2011). Paper discs treated with sterile water and methanol were maintained as controls. Paper discs (6 mm dia) were prepared from Whatman grade 1 filter paper. The plates were incubated for 5 days at 20 ± 2°C, and the areas of inhibition were recorded. The experiment was replicated five times.

### Characterization of crude metabolites by GC-MS

Crude metabolites of the two most effective strains of *B. amyloliquefaciens* VB2 and VB7 were analyzed by GC-MS (Trace GC Ultra DSQ II, Thermo Scientific, Germany) for the detection of active biomolecules responsible for the suppression of *S. sclerotiorum*, as proposed by Dheepa et al. (2016).

### Environmental scanning electron microscope studies

Medium-to-large-sized sclerotial bodies were harvested from the *S. sclerotiorum* culture plates. The sclerotial bodies were plated on nutrient agar medium seeded with *B. amyloliquefaciens* (VB7) @ 100 μl/100 ml. The plates were incubated for 5 days at 20 ± 2°C. An untreated control was also maintained. After incubation, the sclerotial bodies were excised and analyzed under environmental scanning electron microscope (Fei Quanta 200) at the Department of Nano science and Technology, Tamil Nadu Agricultural University, Coimbatore, Tamil Nadu, India. The hyphae from the zone of interaction with antagonist was also analyzed under E-SEM.

To assess the viability of the sclerotial bodies, they were plated on PDA and incubated at 25°C. The experiment was replicated thrice with 30 sclerotia per replication. Healthy sclerotia were also maintained as a control.

### Evaluation of *Bacillus* spp. against *S. sclerotiorum* under protected cultivation

#### Preparation of fungal inoculum

The pathogen was mass multiplied in sand maize medium. Sand maize medium was prepared by thorough mixing of river bed sand and corn meal in the ratio 3:1. After thorough mixing the medium was transferred into autoclavable polypropylene bags and moistened with water. The medium was autoclaved thrice with 12 h interval in between each sterlization. After cooling down, three to five mycelial discs (9 mm dia) of *S. sclerotiorum* were inoculated aseptically and incubated at 20 ± 2°C for 2 months. After 2 months, multiplied inoculum containing white mycelial growth along with sclerotial bodies were taken out and mixed thoroughly. The raised bed prepared for transplanting carnation cuttings were sprinkled with 100 g of inoculum (comprising atleast 100 numbers of sclerotia)/m^2^ area. Following the preparation of sick bed, rooted cuttings were planted.

#### Preparation of bacterial suspension of *Bacillus* spp.

*Bacillus amyloliquefaciens* strains VB2, VB6, VB7, and BSC7; *B. subtilis* strain VB10; and *B. cereus* strain BSC5 were cultured on nutrient agar medium. A loop of 24-hr-old culture of individual strains were inoculated into 1,000 ml of nutrient broth and incubated in an orbital shaker at 150 rpm at room temperature (28 ± 2°C) for 48 h. Later the culture broth was mixed with 1% glycerol (10 ml), 1% Tween 20 (10 ml), and 1% poly vinyl pyrrolidone (10 g) supplied from Sigma-Aldrich. The mixture was incubated in an orbital shaker at 200 rpm for 5 min to ensure uniform mixing. Subsequently, cfu were assessed in nutrient agar medium using the serial dilution technique. The bacterial suspension was prepared comprising minimum of 2.5 × 10^10^ cfu/ml (Dheepa et al., 2016).

### Root-dip and soil drenching of *Bacillus* spp. under protected cultivation

An experiment was conducted during 2013–2014 and 2014–2015 in carnation grown under polyhouses at Nilgiris to assess the bioefficacy of *Bacillus* spp. under protected conditions. Twenty-eight-day-old rooted cuttings of carnations variety charmant pink were dipped in the bacterial suspension of various *Bacillus* strains (2.5 × 10^10^ cfu/ml) at 5 ml/l and planted on beds that were 2.5 ft wide, 30 ft long, and 1 ft high with 15 × 15 cm spacing. The bacterial suspensions of the respective antagonistic bacteria were delivered through soil drenching at monthly intervals at 5 ml/l until the first flush. Carbendazim (50% Wettable Powder) was also included in the treatment and applied at a dose of 2.0 g/l. An untreated control bed was also maintained. Each treatment was replicated thrice, with a 30-ft long bed per replication.

### Observation on percent stem rot incidence and growth parameters

Fifty plants in each treatment were selected at random and tagged to record observations on various traits. All treatments replications were assessed for stem rot incidence, total number of laterals per plant, length of flower stalk, total number of flowers (per m^2^).

### Statistical analysis

Means differences of the treatment were evaluated with ANOVA by using Duncan's Multiple Range-Test at 5% significance (Gomez and Gomez, 1984). All the data were statistically analyzed with IRRISTAT (version. 3/93, Biometrics unit, International Rice Research Institute) and interpreted.

## Results

### Isolation and identification of stem rot pathogen

On PDA, the pathogen produced, white, cottony, mycelial growth with large, black, irregular, sclerotia bodies 7 days after incubation at 20 ± 2°C. Based on the morphological characters, the pathogen was confirmed as *S. sclerotiorum*. Further, the pathogen was also confirmed through sequencing the 18S–28S rRNA genes (KP676452).

### Antifungal activity of *Bacillus* spp. against *Sclerotinia sclerotiorum*

**Figure 1:**
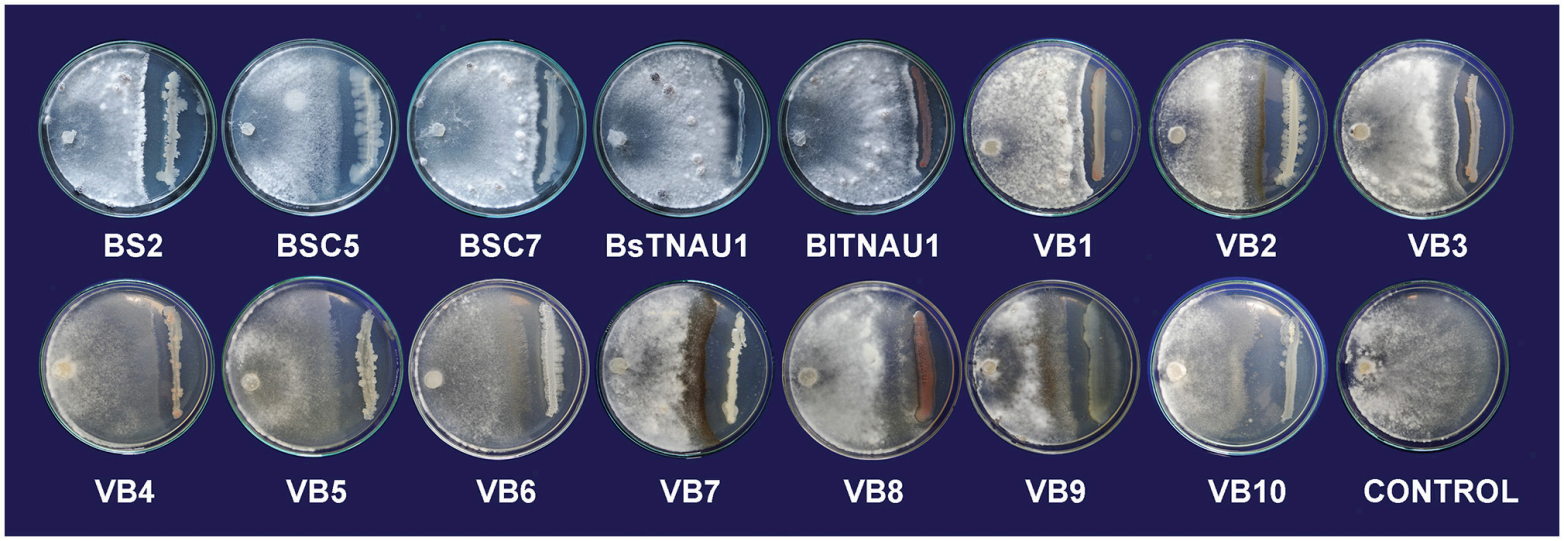
**Suppression of mycelial growth of *Sclerotinia sclerotiorum* by strains of *Bacillus* spp. *in vitro* conditions by using dual culture plate test**.

**Figure 2:**
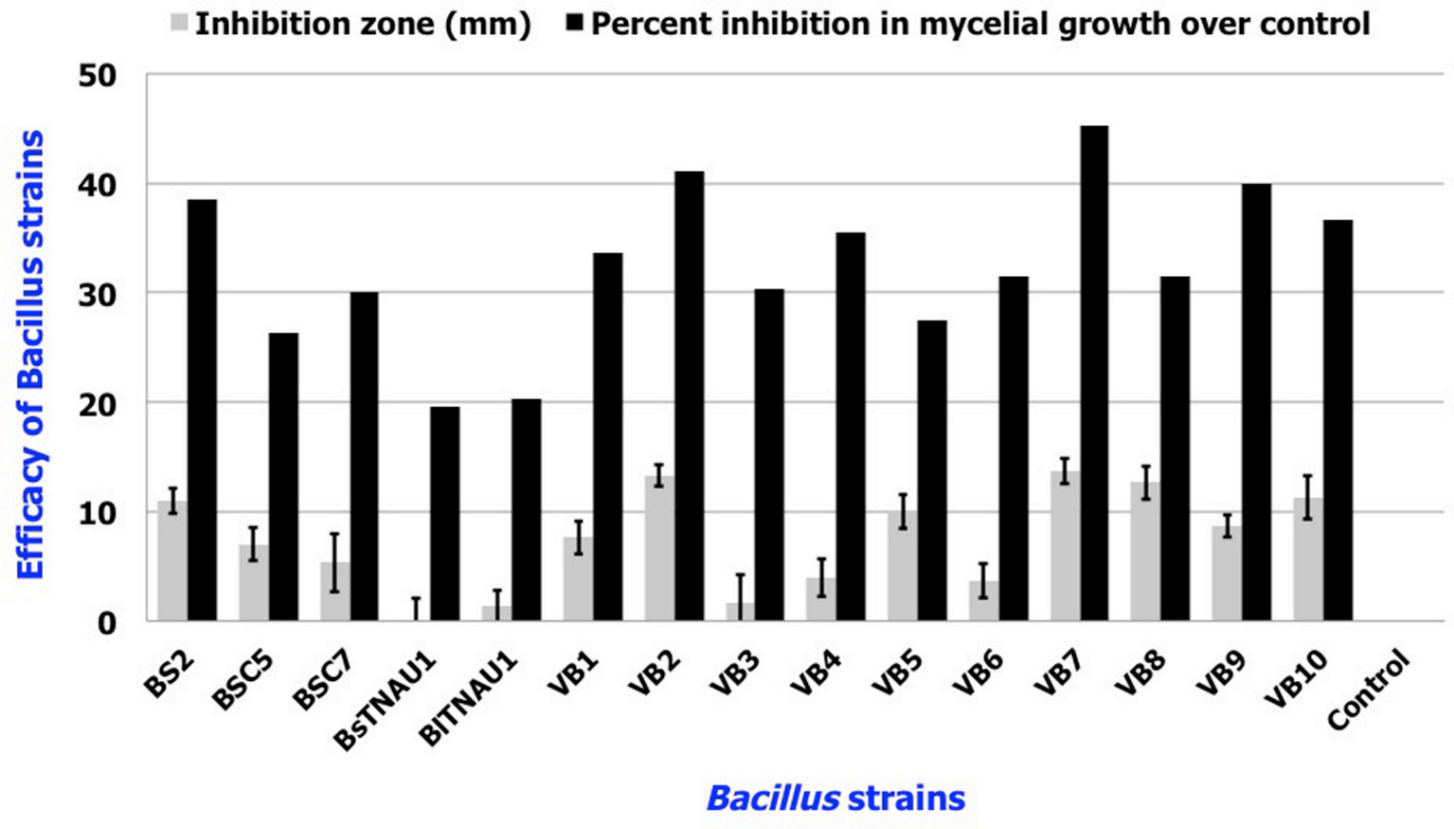
**Antifungal efficacy of *Bacillus* sp. in the suppression of mycelial growth of *S. sclerotiorum in vitro***. Data is presented as mm (inhibition zone) and percentage (Inhibition in mycelial growth over control). Error bars indicate standard deviation obtained from three replicates per treatment. Analysis of variance was performed through DMRT with IRRISTAT (version. 3/93, Biometrics unit, International Rice Research Institute).

**Figure 3:**
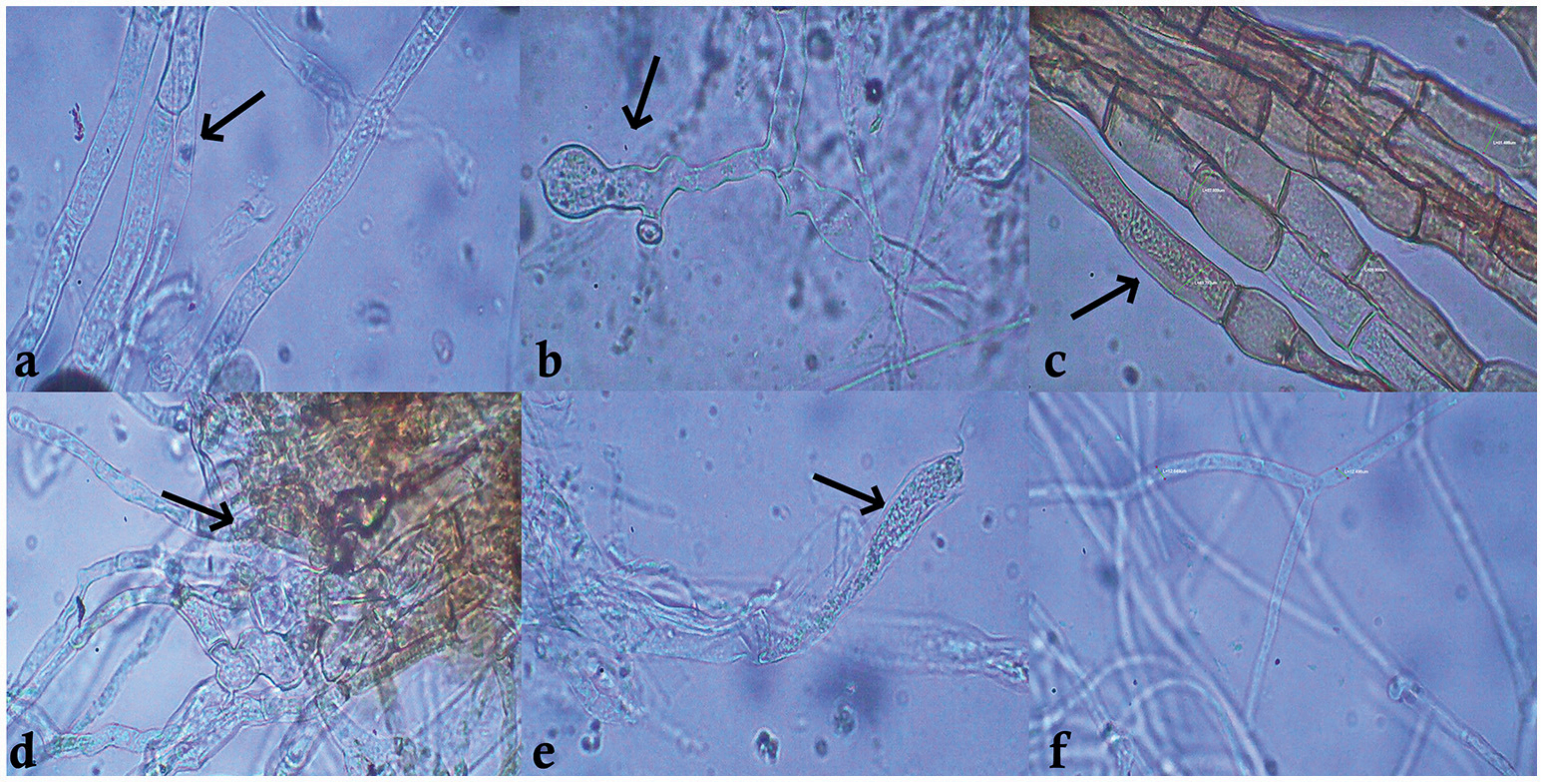
**Mycelial abnormalities of *Sclerotinia sclerotiorum* caused by antimicrobial peptides secreted by *Bacillus amyloliquefaciens* (VB7)**. The black arrows indicate the point of abnormality in the hyphae. **(a–e)** Malformed hyphae of *S. sclerotiorum* exposed to *B. amyoliquefaciens* (VB7). **(f)** Healty hyphae.

Among the 55 strains screened, only 15 strains were found to be effective and were used for further assays. *B. amyloliquefaciens* strain VB7 inhibited the growth of *S. sclerotiorum* up to maximum of 45.19% relative to the control. *B. amyloliquefaciens* (VB2) and *B. subtilis* (VB9) were the next most effective (Figures 1, 2; Table S2). Microscopic observation of mycelia collected from the zone of interaction with *B. amyloliquefaciens* (VB7) revealed that hyphae had malformations such as melanization, swelling, and disintegration (Figure 3).

Viability test of the pathogen at the zone of interaction with antagonist that mycelial plugs of the pathogen collected from the zone of interaction with *B. amyloliquefaciens* (VB7 and VB2) and *B*. *subtilis* (VB9) failed to grow on fresh PDA. This confirmed 100% inhibition of pathogen growth with a single exposure to each antagonist. Subsequently, mycelial plugs exposed to *B. amyloliquefaciens* strain VB6 and *B. subtilis* strain VB10 showed reduced growth compared with control. Mycelial growth of the pathogen was minimal when compared with the untreated wild culture. Mycelial growth of the pathogen was inhibited by 28.17% and 22.20% by strains VB6 and VB10, respectively, relative to the untreated control. In contrast, the mycelial plugs from *B. subtilis* (BS2)-antagonized plates were not suppressed, and the pathogen resumed its normal growth after a short period, indicating that *B. subtilis* (BS2) only temporarily inhibited the pathogen (Table S3; Figure S1).

### Molecular characterization of the effective antagonistic bacteria

The study strains were identified as VB1-*B. subtilis* (KJ603239), VB2-*B. amyloliquefaciens* (KJ603230), VB3-*B. subtilis* (KJ603238), VB4-*B. subtilis* (KJ603231), VB5-*B. amyloliquefaciens* (KJ603232), VB6-*B. amyloliquefaciens* (KJ603233), VB7-*B. amyloliquefaciens* (KJ603234), VB8-*B. amyloliquefaciens* (KJ603235), VB9-*B. subtilis* (KJ603236), and VB10-*B. subtilis* (KJ603237). The sequences showed 89–100% identity with *Bacillus* spp. sequences available in GenBank.

**Figure 4:**
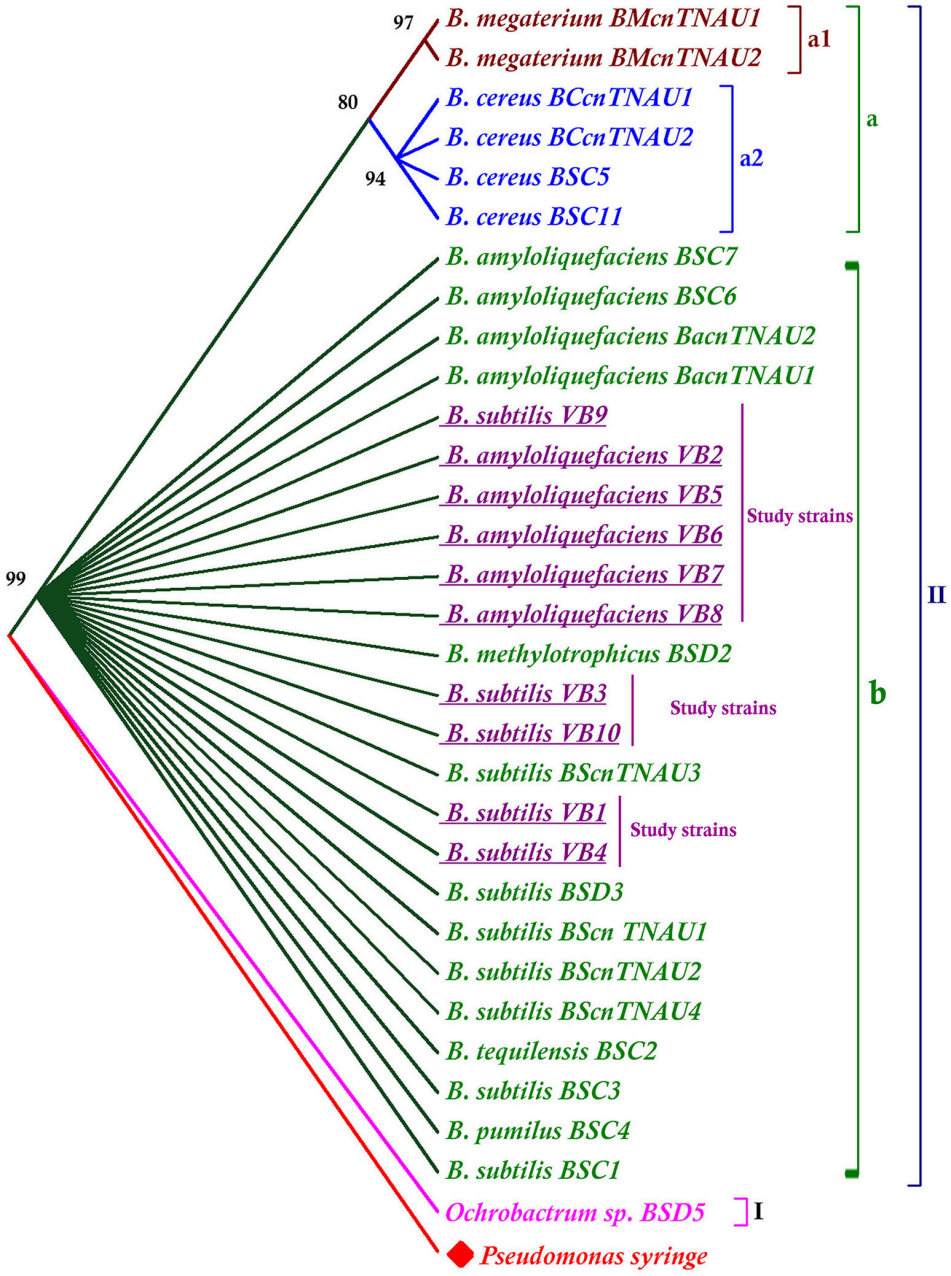
**Phylogenetic tree generated from the 16S rRNA gene sequences of *Bacillus* species**. The neighbor joining tree was computed with Mega 7.0 with 1,000 boot strap replications and a cut off value of 70%.

The phylogenetic tree was rooted with the out group *Pseudomonas syringe*. Apart from the outgroup, the tree was divided into two major clusters. *Ochrobactrum* sp. was grouped separately in one cluster (I). Other *Bacillus* species all together were, grouped into clusterII. Results revealed that *B. subtilis, B. methylotrophicus, B. amyloliquefaciens, B. pumilus, B. tequilensis* were closely related than other species (Figure 4).

### PCR detection of AMP genes

**Figure 5:**
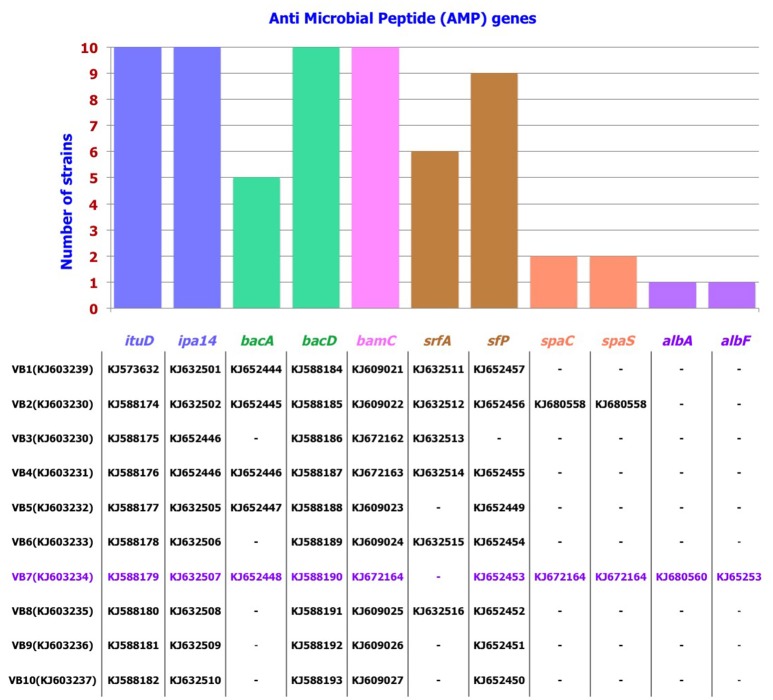
**Distribution pattern of AMP genes in *Bacillus* strains**. Accession number of individual AMP gene pertaining to the strain is listed below.

Among the 10 strains screened, AMP genes related to the antibiotics iturin, bacillomycin, bacilysin, and surfactin were predominant. Following this, antibiotic biosynthesis genes pertaining to mersacidin, ericin, fengycin, subtilin, and subtilosin were also detected. These results were further confirmed by sequencing the AMP genes. Results revealed that these genes showed 79–100% identity with the respective AMP gene sequences available in GenBank. *B. amyloliquefaciens* strain VB7 had the highest number (10) of AMP genes responsible for the biosynthesis of iturin, bacilysin, bacillomycin, surfactin, subtilin, and subtilosin. *B. amyloliquefaciens* strain VB2 followed with nine AMP genes (Figure 5, Figures S2, S3).

### *In vitro* efficacy of volatile compounds from *Bacillus* spp.

**Figure 6:**
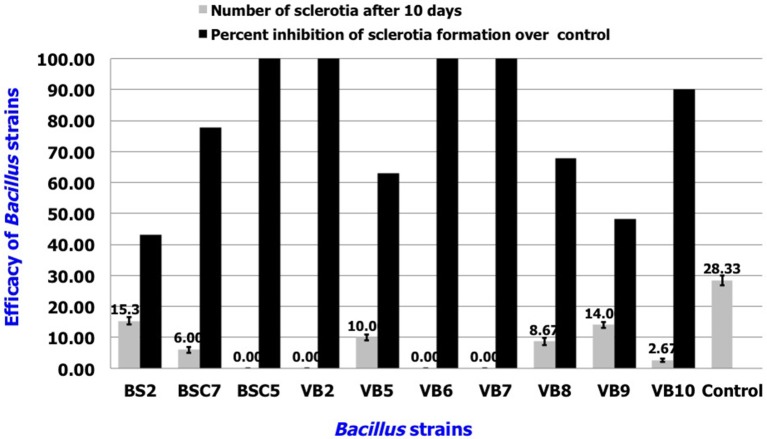
*****In vitro*** efficacy of volatile antimicrobial compounds secreted by ***Bacillus*** sp. in the suppression of sclerotial production**. Data is presented as numbers (sclerotia) and percentage (Inhibition in sclerotial formation over control). Error bars indicate standard deviation obtained from three replicates per treatment. Analysis of variance was performed through DMRT with IRRISTAT (version. 3/93, Biometrics unit, International Rice Research Institute).

Among the tested strains, sclerotial formation was completely inhibited by *B. amyloliquefaciens* strains VB2, VB6, and VB7, as well as *B. cereus* strain BSC5. *B. subtilis* strains BSC7 and VB10 showed the next highest efficacies. Apart from inhibition of sclerotial production, the strains also reduced the density of mycelia compared to the healthy control (Figure 6; Table S4; Figure S4).

### Antifungal activity of crude metabolites against *S. sclerotiorum*

**Figure 7:**
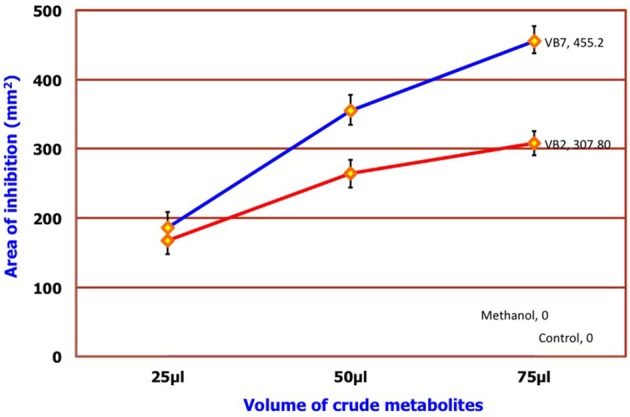
**Efficacy of crude metabolites of *B. amyloliquefaciens***. Data is presented as mm^2^ area. Error bars indicate standard deviation obtained from five replicates per treatment. Analysis of variance was performed through DMRT with IRRISTAT (version. 3/93, Biometrics unit, International Rice Research Institute). Blue line indicates the activity of *B. amyloliquefaciens* (VB7) and red line indicates the activity of *B. amyloliquefaciens* (VB2).

Results of the dual plate assay and partition plate technique indicated that two strains of *B. amyloliquefaciens* (VB2 and VB7) were much more effective in inhibiting the growth of *S. sclerotiorum* than other antagonistic isolates. Seventy-five microliters of crude metabolite extracts of VB7 and VB2 inhibited the mycelial growth of *S. sclerotiorum in vitro* by 455.20 and 307.08 mm^2^, respectively, at 0.1 ppm concentration (Figure 7; Figure S5; Table S5).

### Characterization of crude metabolites by GC/MS

**Figure 8:**
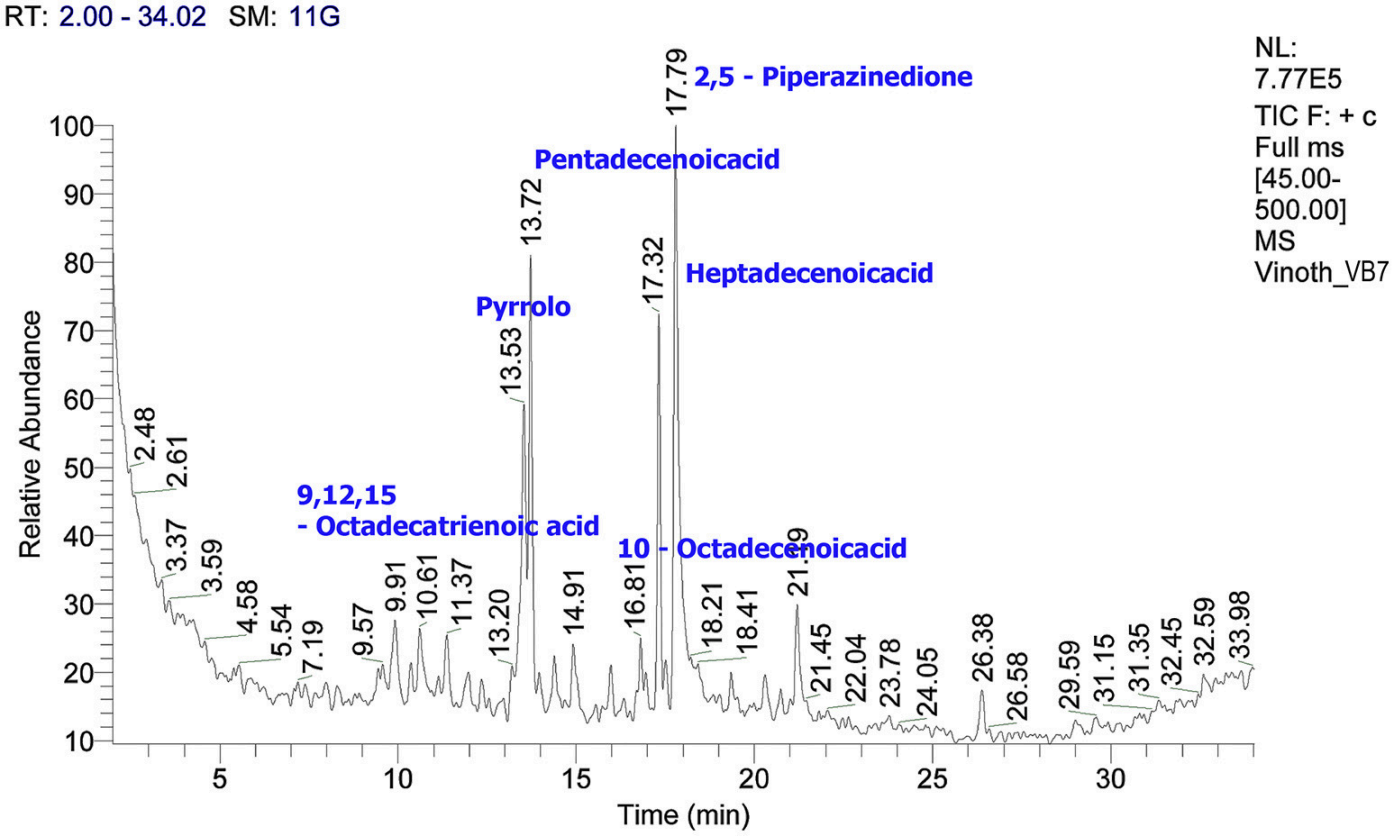
**Total ions chromatogram of crude metabolites of *B. amyloliquefaciens* VB7**. The chromatogram reveals the presence of antifungal metabolites. GC/MS analysis was performed at Dept. of Nano Science and Technology, TNAU, Coimbatore.

**Figure 9:**
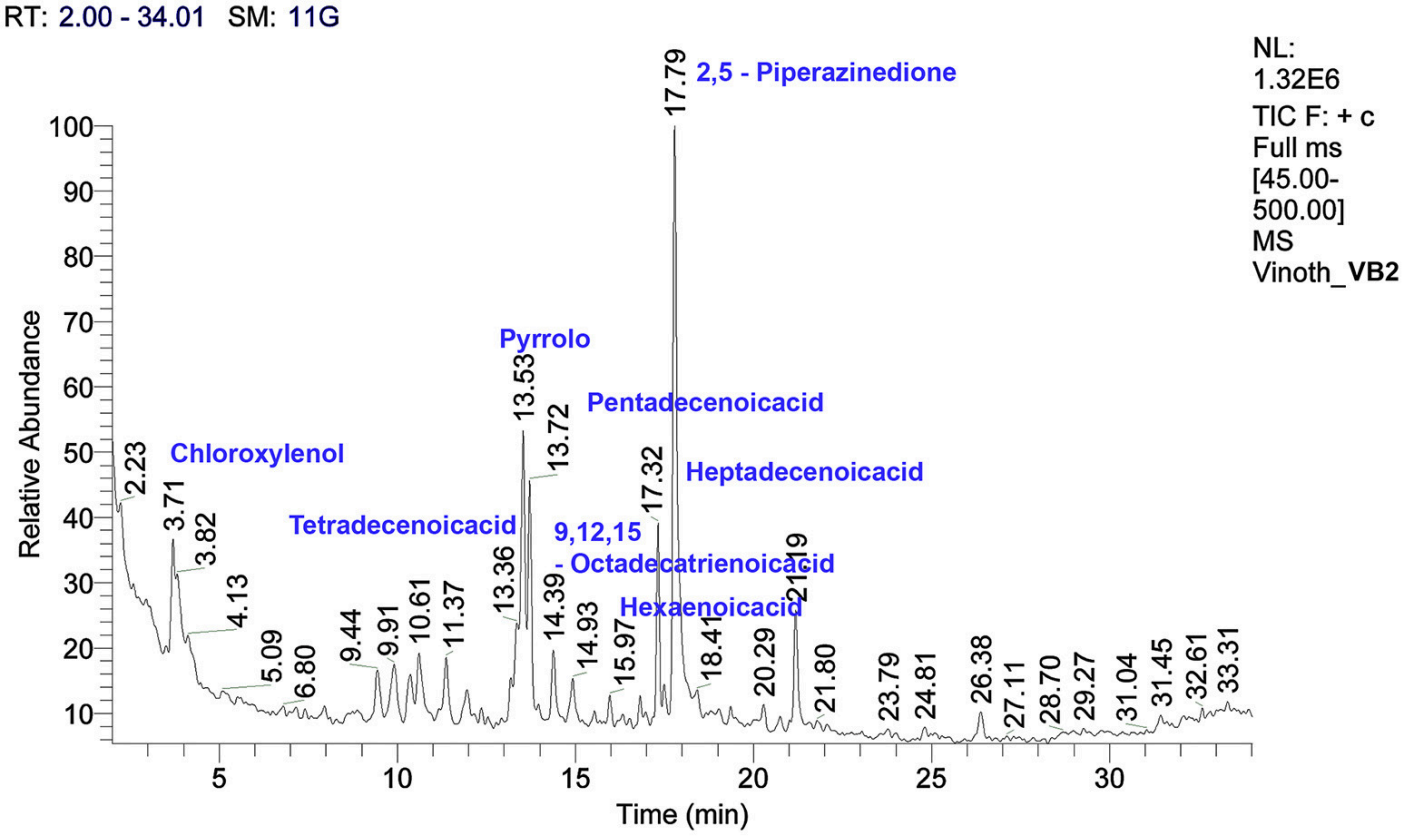
**Total ions chromatogram of crude metabolites of *B. amyloliquefaciens* VB2**. The chromatogram reveals the presence of antifungal metabolites. GC/MS analysis was performed at Dept. of Nano Science and Technology, TNAU, Coimbatore.

The crude metabolites of *B. amyloliquefaciens* strains VB2 and VB7 were analyzed by GC/MS to detect their production of antimicrobial compounds. The compound identity was confirmed using the National Institute of Standards and Technology (NIST) library 2006. Antimicrobial compounds detected in the crude metabolites includes fatty acids an phenols with reported antifungal activity (Figures 8, 9). Most important antifungal compounds detected were, chloroxylenol, pentadecenoicacid, heptadecenoicacid, octadecenoicacid, pyrrolo, and hexadecenoicacid.

### Scanning electron microscopic studies

**Figure 10:**
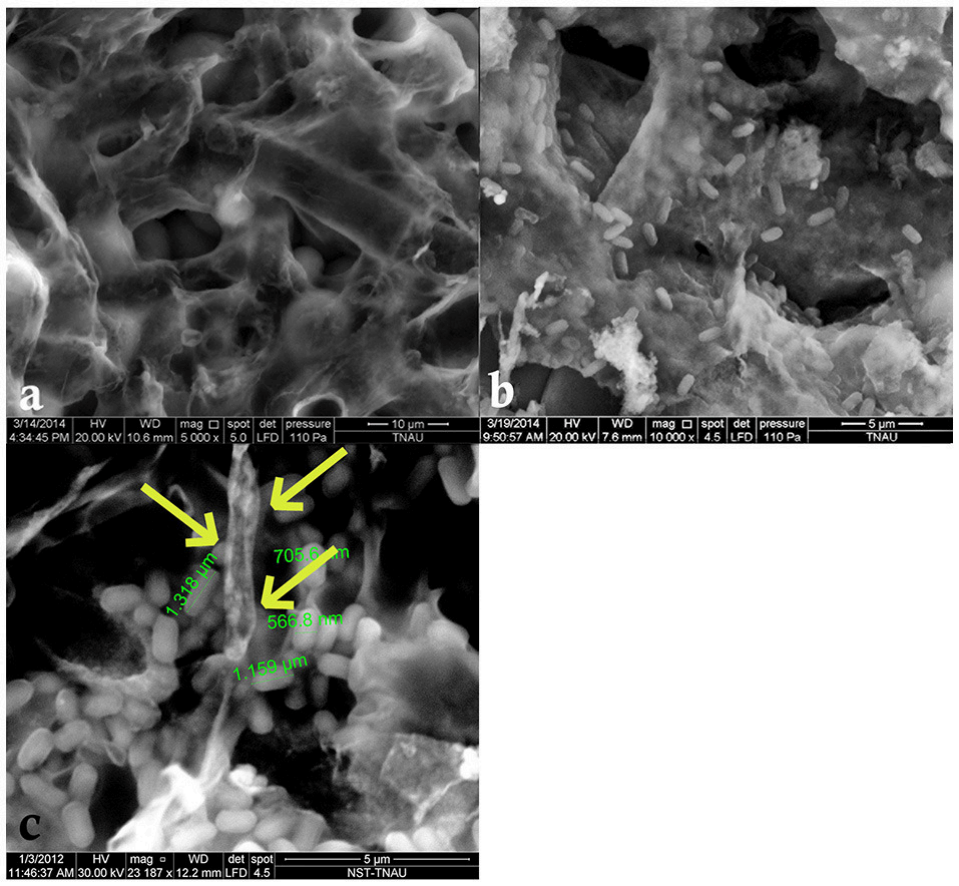
**Scanning electron microscopic observation of antagonized and healthy hyphae and sclerotial bodies. (a)** Scanning Electron Microscopic image of the surface topography of healthy sclerotia of *S. sclerotiorum*. **(b)** Sclerotial surface colonized by *B. amyloliquefaciens* VB7. **(c)** Disintegrated hyphae (indicated with yellow arrow marks) of *S. sclerotiorum* colonized by *B. amyloliquefaciens* (VB7).

Examination of healthy sclerotia of *S. sclerotiorum* by ESEM indicated an undulated topography with numerous, large, deep cavities. In addition, the infected sclerotia revealed that the surface of the sclerotial walls were colonized and *B. amyloliquefaciens* (VB7) survived in the cavities on the surface of the sclerotia (Figure 10). Moreover, 100% inhibition of myceliogenous germination of the sclerotial bodies treated with the suspension of *B. amyloliquefaciens* strain VB7 was observed. In contrast, 100% germination of the sclerotia was observed in the untreated control.

### Effect of *Bacillus* spp. in management of stem rot and the growth promotion in carnation

**Figure 11:**
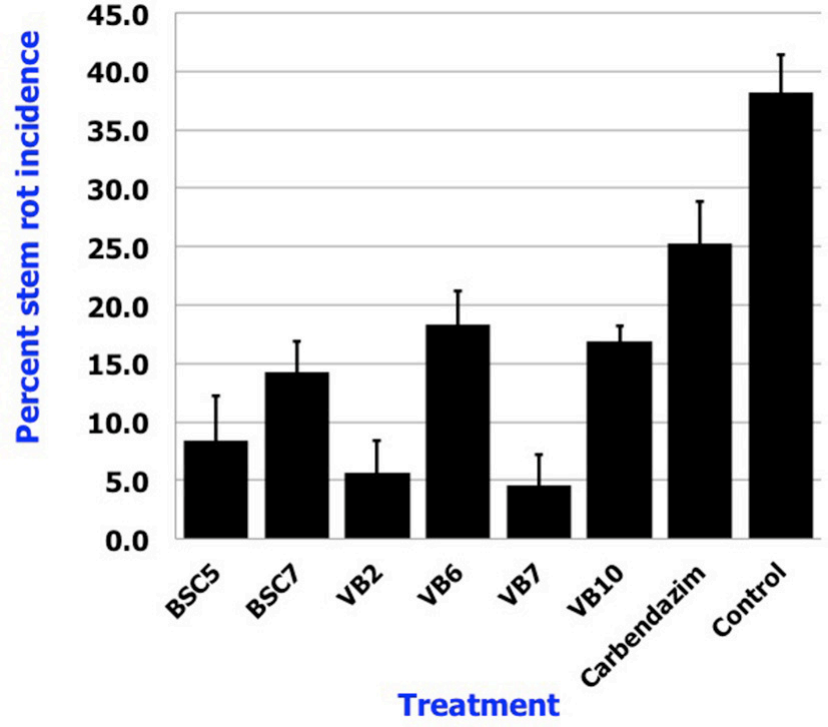
**Efficacy of the *Bacillus* species in the suppression of stem rot of carnations under protected cultivation**. Data is presented as percentage stem rot incidence. Error bars indicate standard deviation obtained from 50 replicates per treatment. The data represents mean value for two season trails conducted (2013–2014 and 2014–2015). Analysis of variance was performed through DMRT with IRRISTAT (version. 3/93, Biometrics unit, International Rice Research Institute).

**Figure 12:**
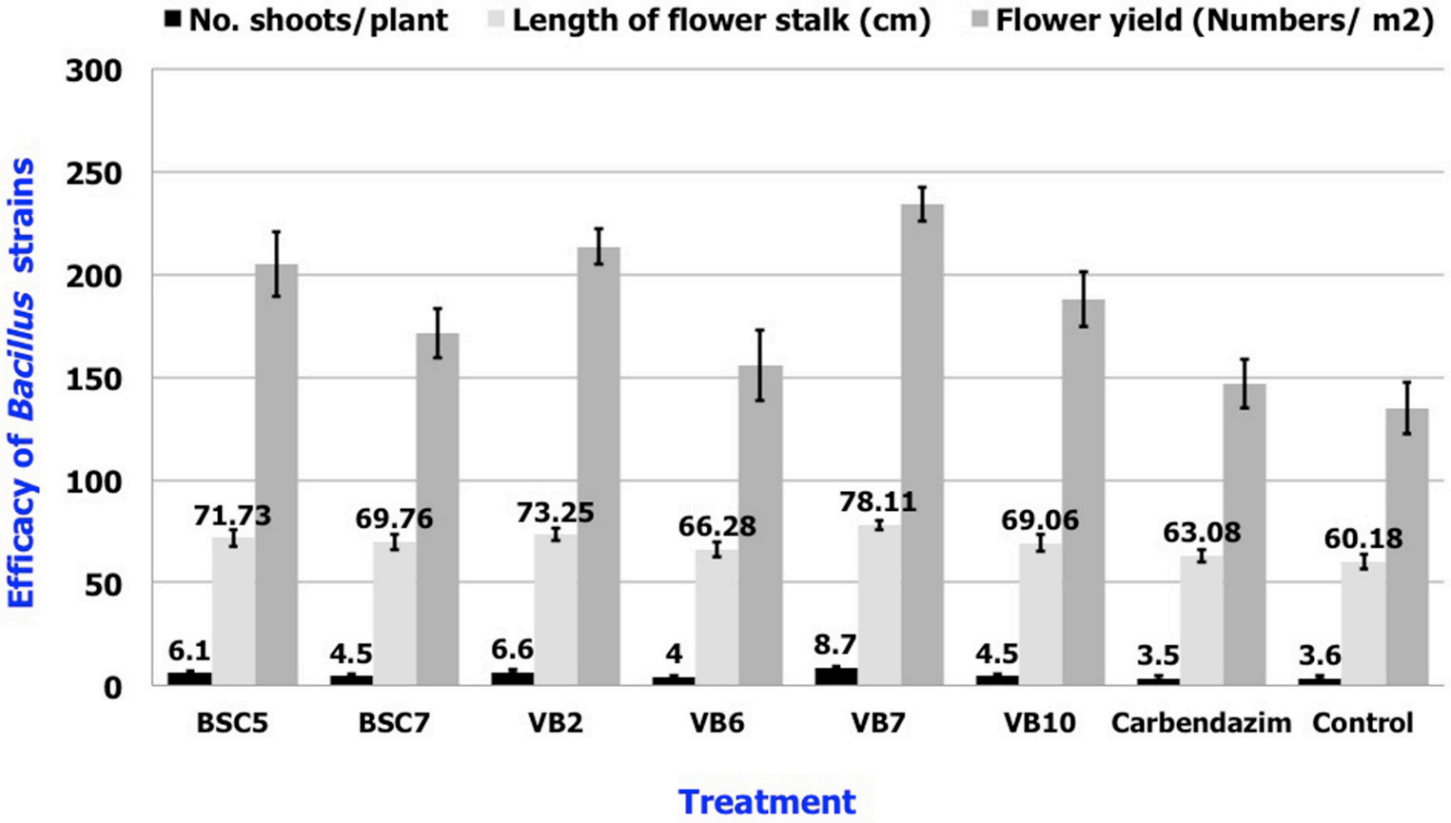
**Efficacy of the *Bacillus* species in the plant growth promotion of carnations under protected cultivation**. Data is presented as numbers (Number of shoots/plant and flower yield/m^2^) and cm (length of flower stalk). Error bars indicate standard deviation obtained from 50 replicates per treatment. The data represents mean value for two season trails conducted (2013–2014 and 2014–2015). Analysis of variance was performed through DMRT with IRRISTAT (version. 3/93, Biometrics unit, International Rice Research Institute).

Results revealed that application of *B. amyloliquefaciens* (VB7) by root dip followed by soil drenching at 0.5% (5 ml/l) resulted in a minimal stem rot incidence of 4.60%, which was reduced 87.9% relative to the control. *B. amyloliquefaciens* (VB2) and *B. cereus* (BSC 5) followed with the next highest efficacies significantly different from each other. For reference, stem rot incidence in the untreated control was 38.24% (Figure 11). Studies on the yield parameters indicated that the mean shoot number, stalk length, and flower yield in the plants treated with *B. amyloliquefaciens* (VB7) were relatively higher than that of the untreated control. Carnation cuttings treated with *B. amyloliquefaciens* (VB7) increased the number of shoots, length of flower stalks, and flower yield compared to the untreated control (Figure 12). *B. amyloliquefaciens* strain VB7 performed better than carbendazim, which reduced stem rot incidence up to 25.20% of. The values are mean of two season trails conducted during 2013–2014 and 2014–2015 (Table S6).

## Discussion

In the present study, we identified effective antagonists with diverse AMP genes that aid in curtailing stem rot of carnations by the production of diverse antimicrobial peptides, including both volatile and non-volatile antimicrobial compounds.

Earlier work by different groups indicated that *B. amyloliquefaciens* inhibited the mycelial growth and sclerotial production of *S. sclerotiorum* (Soylu et al., 2005; Zhang and Xue, 2010). Rahman et al. (2016) reported the efficiency of *B. amyloliquefaciens* in inhibiting mycelial growth of *S. sclerotiorum* causing white mold in mustard. Similarly in our study, *B. amyloliquefaciens* (VB7) effectively inhibited mycelial growth and sclerotial production to a greater extent, significantly different from other treatments. *B. subtilis* (BS2), *B. amyloliquefaciens* (VB2), *B. subtilis* (BS7), and *B. subtilis* (VB9) were the next most effective. Furthermore, the mycelia near to the inhibition zone exposed to *B. amyloliquefaciens* strains VB7 and VB2 were dark brown which was in agreement with previous study by Abdullah et al. (2008). Mycelial plugs from the zone of inhibition next to *B. amyloliquefaciens* (VB2 and VB7) and *B. subtilis* (VB9) inoculations failed to grow on fresh PDA, indicating strong antifungal action of these strains, which resulted in the death of mycelia.

*Bacillus* spp. secrete a wide range of AMPs such as cyclic lipopeptides, which includeiturins, surfactins, fengycins, bacilysin, bacillomycin, mersacidin, and subtilin (Chung et al., 2008; Rajesh Kumar et al., 2014). Our study confirmed the presence of 10 diverse AMP genes, *ituD* and *ipa14* (Iturin), *bacA* and *bacD* (Bacilysin), *bamC* (Bacillomycin), *sfP* (surfactin), *spaC and spas* (subtilin), and *alba* and *albF* (subtilosin), in various *Bacillus* strains. Zhang et al. (2013) reported that bacillomycin secreted by *B. amyloliquefaciens* perturbed the plasma membrane of *Rhizoctonia solani*, abnormalities in conidia and mycelia were also observed. The AMP iturin penetrates cell membranes and creates ion-conducting pore channels, which, in turn, leads to cellular leakage (Ongena and Jacques, 2007). Kenig and Abrahame (1976) reported that bacilysin hinders glucosamine synthesis, which in turn hinders cell wall synthesis. Nasir and Besson (2011) reported that mycosubtilin of *B. subtilis* interacts with the plasma membranes of fungal cells. Hyphal abnormalities were observed on the mycelia of *Pestalotiopsis euginae* treated with cell free extracts of *B. subtilis* that contained iturin and surfactin (Lin et al., 2011). Microscopic studies authenticated this phenomenon. Hyphal abnormalities such as swelling, malformations, melanization, and disintegration of hyphal cells were observed in the hyphae of *S. sclerotiorum* exposed to *B. amyloliquefaciens* (VB7).

In the present investigation, volatiles produced by *B. amyloliquefaciens* completely inhibited sclerotial production by *S. sclerotiorum*. In addition, it also reduced the density of mycelia compared to the uninoculated control, which was in corroboration with earlier study involving by *P. chlororapis* (Fernando et al., 2005).

*Bacillus* species are capable of producing a wide variety of secondary metabolites that are diverse in structure and function. Production of antimicrobial metabolites determines the ability of these species to control plant diseases. Crude extracts of *B. amyloliquefaciens* have been reported to inhibit mycelial growth and sclerotial formation in *S. sclerotiorum* (Ji, 2013). Similarly, in the present study with *B. amyloliquefaciens* (VB7 and VB2), ethyl acetate extracts of crude metabolites reduced mycelial growth. The compounds detected by GC/MS in the crude metabolites of *B. amyloliquefaciens* strains VB2 and VB7 were reported with antimicrobial activity in earlier studies. Chloroxylenol is a chlorine releasing phenolic compound, having ability to disrupt microbial membranes (McDonnell and Russell, 1999). Pentadecenoicacid inhibited the mycelial growth of *Fusarium solani* (Ahmad et al., 2012). Heptadecenoicacid is a satuarated fatty acid reported with antifungal and antibacterial activity by Agoramoorthy et al. (2007). Octadecenoicacid is a hydroxy fatty acid that interacts with microbial membranes. Moreover, the extract inhibited he growth of *Aspergillus niger* and *Penicillium roqueforti* (Black et al., 2013). Pyrrolo is also known to have antifungal activity (Jiang et al., 2014). Hexadecenoic acid has been reported with antifungal activity against *Aspergillus, Penicillium*, and *Fusarium* species (Marrez and Sultan, 2016). In addition to volatile compounds produced by bacteria, volatile plant essential oils are also known to have antifungal activity. Hyphal abnormalities such as swelling, malformations, melanization, and disintegration of hyphal cells were observed in the hyphae and also sclerotia of *S. sclerotiorum* exposed to secondary metabolites and essential oil of fennel plants under light and scanning electron microscopes (Soylu et al., 2007).

Under ESEM, sclerotia of *S. sclerotiorum* were found to be colonized by *B. amyloliquefaciens*. Electron microscopic studies revealed the undulated topography of the sclerotia, with *B. amyloliquefaciens* (VB7) cells residing inside the cavities and present on the surface of sclerotial bodies. Further study indicated the complete inhibition of myceliogenous germination of colonized sclerotia. *B. amyloliquefaciens* (VB7) secreted diverse AMPs, including volatile and non-volatile antibiotics which might have decreased their viability.

Management of plant diseases with methods that minimally affect the environment are desirable. Biocontrol agents can replace chemicals to successfully control several plant diseases. In addition, the extracellular compounds exuded by *Bacillus* species into the rhizosphere promote plant growth and suppress the growth of plant pathogens (Ongena and Jacques, 2007). Cyclic lipopeptide-based AMPs demonstrate a broad spectrum of antimicrobial action against several plant pathogens. Fengycin-producing *B. subtilis* strains S499 and M4 suppressed *F. oxysporum, Rhizoctonia solani*, and *Botrytis cinerea* populations (Ongena et al., 2005). The presence of a greater number of AMP genes has been correlated with the efficiency of the antagonist in controlling plant pathogens (Mora et al., 2011). The combined production of bacilysin, iturin, and mersacidin by *B. subtilis* (ME488) effectively suppressed *Fusarium* wilt of cucumbers and *Phytophthora* blight of peppers (Chung et al., 2008). *Bacillus* mutants that are unable to produce bacillomycin, fengycin, and surfactin lost their ability to control various diseases (Bais et al., 2004).

In the present study, efficacy of *Bacillus* spp. in preventing disease under protected cultivation was significant. *B. amyloliquefaciens* (VB7) curtailed stem rot incidence and promoted plant growth significantly effective compared to any other treatments. In addition, *B. amyloliquefaciens* (VB7) had 10 AMP genes and produced secondary metabolites that had antifungal and antibacterial activity; other strains that were detected had fewer AMP genes. VB7 surpassed other strains of *Bacillus*, indicating the importance of AMP genes in the mitigation of *S. sclerotiorum*.

Study results also indicated that antagonistic *Bacillus* spp. were superior to carbendazim (50% WP), a standard fungicide, included in the treatment. *S. sclerotiorum* has been reported to have developed resistance against carbendazim (Wang et al., 2014). *Sclerotinia* strains that are resistant to carbendazim have been reported to have aminoacid substitutions in the β-tubulin gene (Li et al., 2003). Similarly, many strains of *Sclerotinia* have been reported to resist propineb (Iqbal et al., 2003). Under these circumstances, biological control of carnation stem rot with *Bacillus* strains possessing diversified AMP genes, bestowed with synergistic modes of action, offers a promising alternative.

*Bacillus* species in the natural environment can produce novel beneficial metabolites that promote plant growth and yield (Sharma and Kaur, 2010). *B. amyloliquefaciens* (SQR9) enhanced growth promotion in maize plants (Zhang et al., 2015). Soil application of *B. amyloliquefaciens* promoted plant growth by the production of IAA (Idris et al., 2007) and transposon mutagenesis (Budiharjo et al., 2014). *B. megaterium* have been shown to stimulate cytokinin receptors in plant system through the *CrE1, AHK2*, and *AHK3* genes. These activated receptors induced cytokinin production and promoted plant growth (Ortíz-Castro et al., 2008), elucidating the signaling mechanism between the bacterium and growth promotion. Secretion of plant growth-promoting hormones by bacteria alters the root architecture by leading to the production of more root hairs and lateral roots and subsequently increases water and nutrient uptake. This, in turn, promotes plant growth (Persello-Cartieaux et al., 2003). Similarly, our studies on yield parameters and plant growth promotion, carnation stem cuttings treated with *B. amyloliquefaciens* (VB7) exhibited relatively greater mean shoot number, stalk length, and flower yield compared to untreated controls.

**Figure 13:**
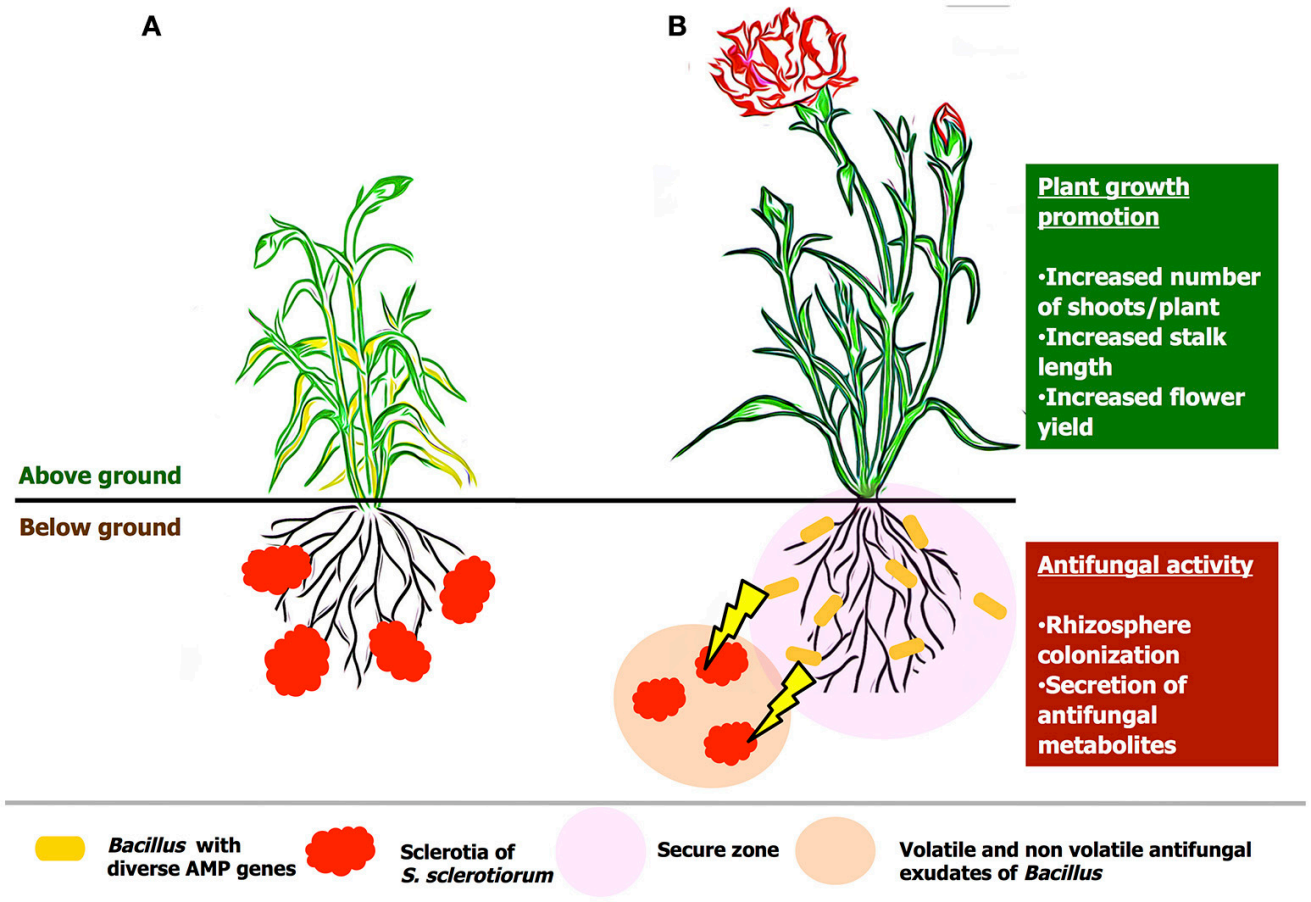
**Hypothetical model on the mode of actions of *Bacillus* species in the management of stem rot of carnations. (A)** an untreated plant, **(B)** plant treated with bacterial suspension of *B. amyloliquefaciens* (VB7). The antagonist colonize the rhizosphere region. Further, prevents the establishment of the pathogen by the hyper-parasitism and antifungal activity by the secretion of volatile and non-volatile compounds.

*B. amyloliquefaciens* VB7 is an effective beneficial bacterial antagonists with multifaceted mode of action (Figure 13). The mode of action includes, competitive colonization of rhizosphere, secretion of volatile and non-volatile anti-fungal compounds as well anti-microbial peptides. Soil application of *B. amyloliquefaciens* VB7 was effective in reducing the stem rot incidence as well-promoted plant growth and yield. Moreover, other efficiency of other strains including *B. amyloliquefaiens* (VB2) and *B. subtilis* (VB9) is also notable in inhibiting the development of the pathogen. In this constraint, future studies in the utilization of *Bacillus* species with diverse anti-microbial peptide genes would be a scope for the reduction various fungal diseases across crop plants.

## Author contributions

SV performed the laboratory experiments, evaluation under protected cultivation, statistical analysis, data recording, and manuscript write-up. SN designed the experiment, supervised the study, mobilized the consumable for the study. PR and VM co-supervised the study. All authors proofread and reviewed the manuscript.

### Conflict of interest statement

The authors declare that the research was conducted in the absence of any commercial or financial relationships that could be construed as a potential conflict of interest.
