# Supplementary material for: Biocontrol Potentials of Antimicrobial Peptide Producing Bacillus Species: Multifaceted Antagonists for the Management of Stem Rot of Carnation Caused by Sclerotinia sclerotiorum
Source: Front Microbiol. 2017 Mar 24;8:446. doi: 10.3389/fmicb.2017.00446 (PMC5364326; doi:10.3389/fmicb.2017.00446)
Supplement: Supplementary file 1 [file Table1.DOCX]

**Table S1. List of primer pairs used for the detection of AMP genes**

| **S.No** | **Antibiotic** | **Pertaining gene** | **Primer sequence** | **Product size bp** | **Reference** |
| --- | --- | --- | --- | --- | --- |
| 1. | Iturin | ***ituA*** | ITUD1F 5’-GATGCGATCTCCTTGGATGT-3’  ITUD1R 5’-ATCGTCATGTGCTGCTTGAG-3’ | 647 | Ramarathnam, 2007 |
|  |  | ***ituC*** | ITUC-F1 5′-CCCCCTCGGTCAAGTGAATA  ITUC-R1 5′-TTGGTTAAGCCCTGATGCTC | 594 | Chung *et al.,* 2008 |
|  |  | ***ituD*** | ITUD-F1 5’-TTG AAYGTCAGYGCSCCTTT-3’  ITUD-R1 5’-TGCG MAAATGGSGT CGT-3’ | 482 | Chung *et al.,* 2008 |
|  |  | ***ipa 14*** | ipa14f 5’-ATG AAA ATT TAC GGA GTA TA-3’  ipa14r 5’-TTA TAA CAG CTC TTC ATA CG-3’ | 675 | Hsieh*et al.,* 2008 |
| 2. | Bacillomycin | ***bamC*** | BAMC-F1 5′-AGTAAATGAACGCGCCAATC-3’  BAMC-R1 5′-CCCTCTCCTGCCACATAGAG-3’ | 957 | Chung *et al.,* 2008 |
| 3. | Bacilysin | ***bacAB*** | BACAB-F1 5′-CTTCTCCAAGGGGTGAACAG-3’  BACAB-R1 5′-TGTAGGTTTCACCGGCTTTC-3’ | 815 | Chung *et al.,* 2008 |
|  |  | ***bacD*** | BACD-F1 5′-AAAAACAGTATTGGTYATCGCTGA-3’  BACD-R1 5′-CCATGATGCCTTCKATRCTGAT-3’ | 749 | Chung *et al.,* 2008 |
| 4. | Mersacidin | ***mrsA*** | MRSA-F1 5′-GGGTATATGCGGTATAAACTTATG-3’  MRSA-R1 5′-GTTTCCCCAATGATTTACCCTC-3’ | 597 | Altena *et al.,* 2000 |
| 5. | Fengycin | ***fenA*** | FENCEA-F1 5′-CCCATCCGACYGTAGAAG-3’  FENCEA-R1 5′-GTGTAAGCRGCAAGYAGCAC-3’ | 820 | Chung *et al.,* 2008 |
|  |  | ***fenB*** | FENB-F15′-CCTGGAGAAAGAATATACCGTACCY-3’  FENB-R1 5′-GCTGGTTCAGTTKGATCACAT-3’ | 670 | Chung *et al.,* 2008 |
|  |  | ***fenC*** | FENCEA-F1 5′-CCCATCCGACYGTAGAAG-3’  FENCEA-R1 5′-GTGTAAGCRGCAAGYAGCAC-3’ | 820 | Chung *et al.,* 2008 |
|  |  | ***fend*** | FEND1F 5’-TTTGGCAGCAGGAGAAGTTT3’  FEND1 R 5’-GCTGTCCGTTCTGCTTTTTC3’ | 974 | Chung *et al.,* 2008 |
|  |  | ***fenE*** | FENCEA-F1 5′-CCCATCCGACYGTAGAAG-3’  FENCEA-R1 5′-GTGTAAGCRGCAAGYAGCAC-3’ | 820 | Chung *et al.,* 2008 |
| 6. | Surfactin | ***srfA*** | SRFA-F1 5′-AGAGCACATTGAGCGTTACAAA-3’  SRFA-R1 5′-CAGCATCTCGTTCAACTTTCAC-3’ | 626 | Chung *et al.,* 2008 |
|  |  | ***Sfp*** | SFP-F1 5’TTTGGCAGCAGGAGAAGTTT3’  SFP-R1 5’GCTGTCCGTTCTGCTTTTTC3’ | 675 | Hsieh *et al.,* 2004 |
| 7. | Mycosubtilin | ***mycC*** | MYCC-F15′-AATCAATTGGCACGAACCTT-3’  MYCC-R1 5′-ATCGCCCGTTTTGTACATTC-3’ | 1026 | Chung *et al.,* 2008 |
| 8. | Ericin | ***eriB*** | SPAB-ERIB-F1 5′-GCACAGATGGAAAATCTGAAG-3’  SPAB-ERIB-R1 5′-GAAAATTGCTCCCCAAATGA-3’ | 688 | Chung *et al.,* 2008 |
| 9. | Subtilin | ***spaC*** | SPACS-F1 5′-CCGGACAGGAGTATTTTAAGGA-3’  SPACS-R1 5′-GCAGTTACAAGTTAGTGTTTGAAGGA-3’ | 460 | Chung *et al.,* 2008 |
|  |  | ***spaS*** | SPACS-F1 5′-CCGGACAGGAGTATTTTAAGGA-3’  SPACS-R1 5′-GCAGTTACAAGTTAGTGTTTGAAGGA-3’ | 460 | Chung *et al.,* 2008 |
| 10. | Subtilosin | ***alba*** | ALBA-F1 5′-TTGTTTATAGAGCAGATGTTTCCA-3’  ALBA-R1 5′-GGCTCTCTTTTCGCATGAGT-3’ | 625 | Chung *et al.,* 2008 |
|  |  | ***albF*** | ALBFF1 5’-TCAACAGCTGGATGAACGAAC-3’ALBF R1 5’-AGGCGGTAYGTTTGCTGWATCT-3’ | 888 | Chung *et al.,* 2008 |
