# Supplementary material for: Biocontrol Potentials of Antimicrobial Peptide Producing Bacillus Species: Multifaceted Antagonists for the Management of Stem Rot of Carnation Caused by Sclerotinia sclerotiorum
Source: Front Microbiol. 2017 Mar 24;8:446. doi: 10.3389/fmicb.2017.00446 (PMC5364326; doi:10.3389/fmicb.2017.00446)
Supplement: Supplementary file 2 [file Table2.DOCX]

**Table S2. Antifungal activity of *Bacillus* spp., against *S. sclerotiorum in vitro***

| **S.No** | **Isolates** | **Mycelial growth (mm)*** | **Percent inhibition over control** | **Inhibition zone**  **(mm)*** |
| --- | --- | --- | --- | --- |
| 1. | *B.subtilis* (BS2) - JN873298 | 55.33 a-d | 38.52 | 12.33 ab |
| 2. | *B. cereus* (BSC5) - JX036520 | 66.33 fgh | 26.30 | 7.67 de |
| 3. | *B. amyloliquefaciens* (BSC7) -JX036522 | 63.00 def | 30.00 | 5.33 ef |
| 4. | *B. subtilis* (BsTNAU1) - KC540800 | 72.33 g-j | 19.63 | 0.67 i |
| 5. | *B. licheniformis* (BlTNAU1) - KC540811 | 71.67 ghi | 20.37 | 2.33 h |
| 6. | *B. subtilis* (VB1) -KJ603239 | 59.67 b-f | 33.70 | 7.67 de |
| 7. | *B. amyloliquefaciens* (VB2) -KJ603230 | 53.00 ab | 41.11 | 12.00 abc |
| 8. | *B. subtilis* (VB3) - KJ603238 | 62.67 def | 30.37 | 3.00 gh |
| 9. | *B. subtilis* (VB4) - KJ603231 | 58.00 b-f | 35.56 | 4.33 fg |
| 10. | *B. amyloliquefaciens* (VB5) -KJ603232 | 65.33 efg | 27.41 | 10.00 bcd |
| 11. | *B. amyloliquefaciens* (VB6) -KJ603233 | 61.67 c-f | 31.48 | 4.67 fg |
| 12. | *B. amyloliquefaciens* (VB7) - KJ603234 | 49.33 a | 45.19 | 15.33 a |
| 13. | *B. amyloliquefaciens* (VB8) - KJ603235 | 61.67 c-f | 31.48 | 14.00 a |
| 14. | *B. subtilis* (VB9) - KJ603236 | 54.00 abc | 40.00 | 8.67 cd |
| 15. | *B. subtilis* (VB10) - KJ603237 | 57.00 a-e | 36.67 | 11.67 abc |
| 16. | VB11 – un identified | 76.33 i-l | 15.19 | - |
| 17. | VB12 – un identified | 76.33 i-l | 15.19 | - |
| 18. | VB13 – un identified | 79.00 i-n | 12.22 | - |
| 19. | VB14 – un identified | 75.00 ijk | 16.67 | - |
| 20. | VB15 – un identified | 81.33 k-o | 9.63 | - |
| 21. | VB16 – un identified | 83.67 m-q | 7.04 | - |
| 22. | VB17 – un identified | 88.00 pq | 2.22 | - |
| 23. | VB18 – un identified | 87.33 opq | 2.96 | - |
| 24. | VB19 – un identified | 74.67 ijk | 17.04 | - |
| 25. | VB20 – un identified | 89.00 pq | 1.11 | - |
| 26. | VB21 – un identified | 89.00 pq | 1.11 | - |
| 27. | VB22 – un identified | 89.00 pq | 1.11 | - |
| 28. | VB23 – un identified | 89.00 pq | 1.11 | - |
| 29. | VB24 – un identified | 84.00 m-q | 6.67 | - |
| 30. | VB25 – un identified | 88.67 pq | 1.48 | - |
| 31. | VB26 – un identified | 89.00 pq | 1.11 | - |
| 32. | VB27 – un identified | 85.00 n-q | 5.56 | - |
| 33. | VB28 – un identified | 79.67 j-n | 11.48 | - |
| 34. | VB29 – un identified | 74.00 h-k | 17.78 | - |
| 35. | VB30 – un identified | 88.67 pq | 1.48 | - |
| 36. | VB31 – un identified | 89.00 pq | 1.11 | - |
| 37. | VB32 – un identified | 82.67 l-p | 8.15 | - |
| 38. | VB33 – un identified | 74.00 h-k | 17.78 | - |
| 39. | VB34 – un identified | 76.00 i-l | 15.56 | - |
| 40. | VB35 – un identified | 88.33 pq | 1.85 | - |
| 41. | VB36 – un identified | 89.00 pq | 1.11 | - |
| 42. | VB37 – un identified | 88.67 pq | 1.48 | - |
| 43. | VB38 – un identified | 74.00 h-k | 17.78 | - |
| 44. | VB39 – un identified | 77.33 i-m | 14.07 | - |
| 45. | VB40 – un identified | 73.67 h-k | 18.15 | - |
| 46. | VB41 – un identified | 75.67 i-l | 15.93 | - |
| 47. | VB42 – un identified | 73.33 hij | 18.52 | - |
| 48. | VB43 – un identified | 79.33 j-n | 11.85 | - |
| 49. | VB44 – un identified | 84.00 m-q | 6.67 | - |
| 50. | VB45 – un identified | 78.67 i-n | 12.59 | - |
| 51. | VB46 – un identified | 88.00 pq | 2.22 | - |
| 52. | VB47 – un identified | 84.33 m-q | 6.30 | - |
| 53. | VB48 – un identified | 88.00 pq | 2.22 | - |
| 54. | VB49 – un identified | 88.67 pq | 1.48 | - |
| 55. | VB50 – un identified | 89.00 pq | 1.11 | - |
| 56. | Control | 89.33 q | - | - |

*Values are mean of three replications.

In a column, means followed by a common letter are not significantly different at the 5% level by Duncan’s Multiple Range Test
