# Supplementary material for: Biocontrol Potentials of Antimicrobial Peptide Producing Bacillus Species: Multifaceted Antagonists for the Management of Stem Rot of Carnation Caused by Sclerotinia sclerotiorum
Source: Front Microbiol. 2017 Mar 24;8:446. doi: 10.3389/fmicb.2017.00446 (PMC5364326; doi:10.3389/fmicb.2017.00446)
Supplement: Supplementary file 3 [file Table3.DOCX]

**Table S3.** ***In vitro* suppression of mycelial growth of *S. sclerotiorum* by different strains of *Bacillus* spp.**

| **S.No** | **Isolates** | **Mycelial growth (mm)*** | **Percent Inhibition of mycelial growth over control** |
| --- | --- | --- | --- |
| 1 | *B. subtilis* (BS2) | 90.00 d | 0.00 |
| 2 | *B. amyloliquefaciens* (VB2) | 0.00 a | 100.00 |
| 3 | *B. amyloliquefaciens* (VB6) | 64.67 b | 28.17 |
| 4 | *B. amyloliquefaciens* (VB7) | 0.00 a | 100.00 |
| 5 | *B. subtilis* (VB9) | 0.00 a | 100.00 |
| 6 | *B. subtilis* (VB10) | 70.00 c | 22.20 |
| 7 | Control | 90.00 d | - |

*Values are mean of three replications.

In a column, means followed by a common letter are not significantly different at the 5% level by Duncan’s Multiple Range Test
