# Supplementary material for: Biocontrol Potentials of Antimicrobial Peptide Producing Bacillus Species: Multifaceted Antagonists for the Management of Stem Rot of Carnation Caused by Sclerotinia sclerotiorum
Source: Front Microbiol. 2017 Mar 24;8:446. doi: 10.3389/fmicb.2017.00446 (PMC5364326; doi:10.3389/fmicb.2017.00446)
Supplement: Supplementary file 4 [file Table4.DOCX]

**Table S4. Suppression of sclerotial formation by volatile compounds of *Bacillus* spp. under divided plate technique**

| **S.No** | **Isolate** | **Number of sclerotia after 10 days*** | **Percent inhibition of sclerotia formation over control** |
| --- | --- | --- | --- |
| 1. | *B.subtilis* (BS2) | 15.33 e | 77.78 |
| 2. | *B.subtilis* (BSC7) | 6.00 c | 100.00 |
| 3. | *B. cereus* (BSC5) | 0.00 a | 100.00 |
| 4. | *B.amyloliquefaciens* (VB2) | 0.00 a | 62.96 |
| 5. | *B*. *amyloliquefaciens* (VB5) | 10.00 d | 100.00 |
| 6. | *B.amyloliquefaciens* (VB6) | 0.00 a | 100.00 |
| 7. | *B.amyloliquefaciens* (VB7) | 0.00 a | 67.90 |
| 8. | *B.amyloliquefaciens* (VB8) | 8.67 d | 48.15 |
| 9. | *B.subtilis* (VB9) | 14.00 e | 90.12 |
| 10. | *B.subtilis* (VB10) | 2.67 b | 77.78 |
| 11. | Control | 28.33 f | - |

*Values are mean of three replications.

In a column, means followed by a common letter are not significantly different at the 5% level by Duncan’s Multiple Range Test
