# Supplementary material for: Biocontrol Potentials of Antimicrobial Peptide Producing Bacillus Species: Multifaceted Antagonists for the Management of Stem Rot of Carnation Caused by Sclerotinia sclerotiorum
Source: Front Microbiol. 2017 Mar 24;8:446. doi: 10.3389/fmicb.2017.00446 (PMC5364326; doi:10.3389/fmicb.2017.00446)
Supplement: Supplementary file 5 [file Table5.DOCX]

**Table S5. Antifungal activity of crude metabolites of *B. amyloliquefaciens* strains against mycelial growth of *S. sclerotiorum***

| **S.No** | **Isolate** | **Area of inhibition (mm^2^)*** | | |
| --- | --- | --- | --- | --- |
|  |  | **25µl** | **50µl** | **75µl** |
| 1. | *B. amyloliquefaciens*(VB2) | 167.40 a | 264.00 b | 307.08 b |
| 2. | *B. amyloliquefaciens* (VB7) | 186.00 a | 354.60 a | 455.20 a |
| 3. | Methanol | 0.00 c | 0.00 c | 0.00 c |
| 4. | Untreated control | 0.00 c | 0.00 c | 0.00 c |

*Values are mean of five replications

Means followed by a common letter are not significantly different at 5% level by Duncan’s Multiple Range Test
