# Supplementary material for: Biocontrol Potentials of Antimicrobial Peptide Producing Bacillus Species: Multifaceted Antagonists for the Management of Stem Rot of Carnation Caused by Sclerotinia sclerotiorum
Source: Front Microbiol. 2017 Mar 24;8:446. doi: 10.3389/fmicb.2017.00446 (PMC5364326; doi:10.3389/fmicb.2017.00446)
Supplement: Supplementary file 6 [file Table6.DOCX]

**Table S6. Suppression of stem rot disease incited by *S. sclerotiorum* on carnation (cv. Charmant pink) by different strains of *Bacillus* spp., under protected cultivation (2013-14 and 2014-15)**

| **Treatment – Root dipping followed by soil drenching**** | | **Percentage stem rot incidence*** | **No. shoots/plant** | **Length of flower stalk* (cm)** | **Flower yield/ m^2^** |
| --- | --- | --- | --- | --- | --- |
| T1 | *B. cereus* (BSC5) @ 5ml/litre | 8.34 c | 6.10 c | 71.73 c | 204.86 c |
| T2 | *B.amyloliquefaciens* (BSC7) @ 5ml/litre | 14.20 d | 4.50 d | 69.76 d | 171.24 e |
| T3 | *B. amyloliquefaciens* (VB2) @ 5ml/litre | 5.68 b | 6.60 b | 73.25 b | 213.46 b |
| T4 | *B. amyloliquefaciens* (VB6) @ 5ml/litre | 18.38 f | 4.00 e | 66.28 e | 155.86 f |
| T5 | *B. amyloliquefaciens* (VB7) @ 5ml/litre | 4.60 a | 8.70 a | 78.11 a | 234.18 a |
| T6 | *B. subtilis* (VB10) @ 5ml/litre | 16.90 e | 4.50 d | 69.06 d | 188.06 d |
| T7 | Carbendazim 50% (WP) @ 2.0g/litre | 25.20 g | 3.50 f | 63.08 f | 147.00 g |
| T8 | Control | 38.24 h | 3.60 f | 60.18 g | 134.86 h |

*Values are mean of three replications

** Soil drenching was given once in a month

Means followed by a common letter are not significantly different at 5% level by Duncan’s Multiple Range Test
