# Supplementary figures and images for: Biocontrol Potentials of Antimicrobial Peptide Producing Bacillus Species: Multifaceted Antagonists for the Management of Stem Rot of Carnation Caused by Sclerotinia sclerotiorum
Source: Front Microbiol. 2017 Mar 24;8:446. doi: 10.3389/fmicb.2017.00446 (PMC5364326; doi:10.3389/fmicb.2017.00446)

**Figure S1. Viability of *Sclerotinia sclerotiorum* exposed to *Bacillus* spp. *in vitro***

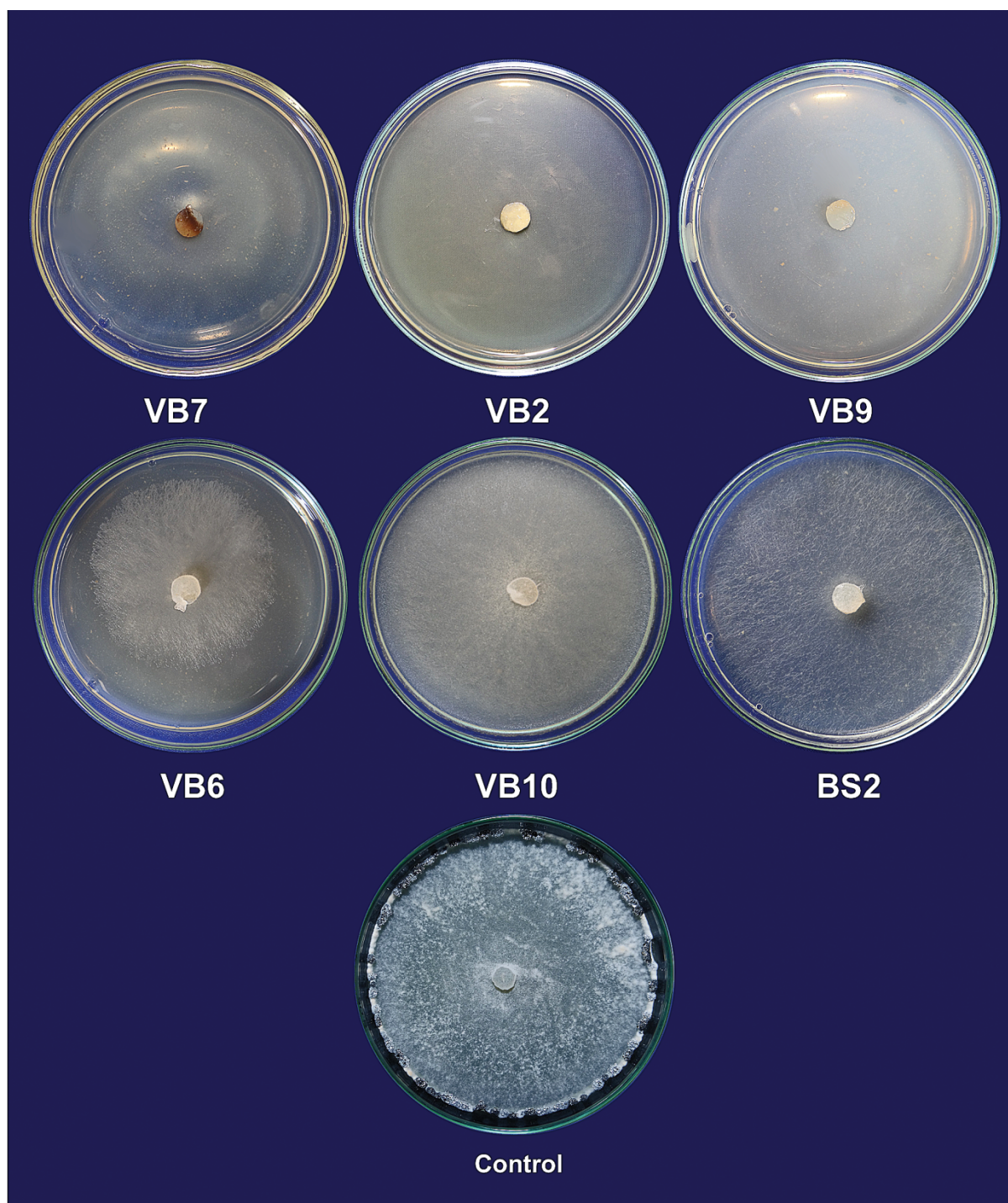

Supplement: Supplementary file 7 [file Image1.PDF]
