# Supplementary material for: Biocontrol Potentials of Antimicrobial Peptide Producing Bacillus Species: Multifaceted Antagonists for the Management of Stem Rot of Carnation Caused by Sclerotinia sclerotiorum
Source: Front Microbiol. 2017 Mar 24;8:446. doi: 10.3389/fmicb.2017.00446 (PMC5364326; doi:10.3389/fmicb.2017.00446)

Figure S2. Agarose gel electrophoresis of AMP genes related to iturin, bacilysin, bacillomycin and ericin antibiotics

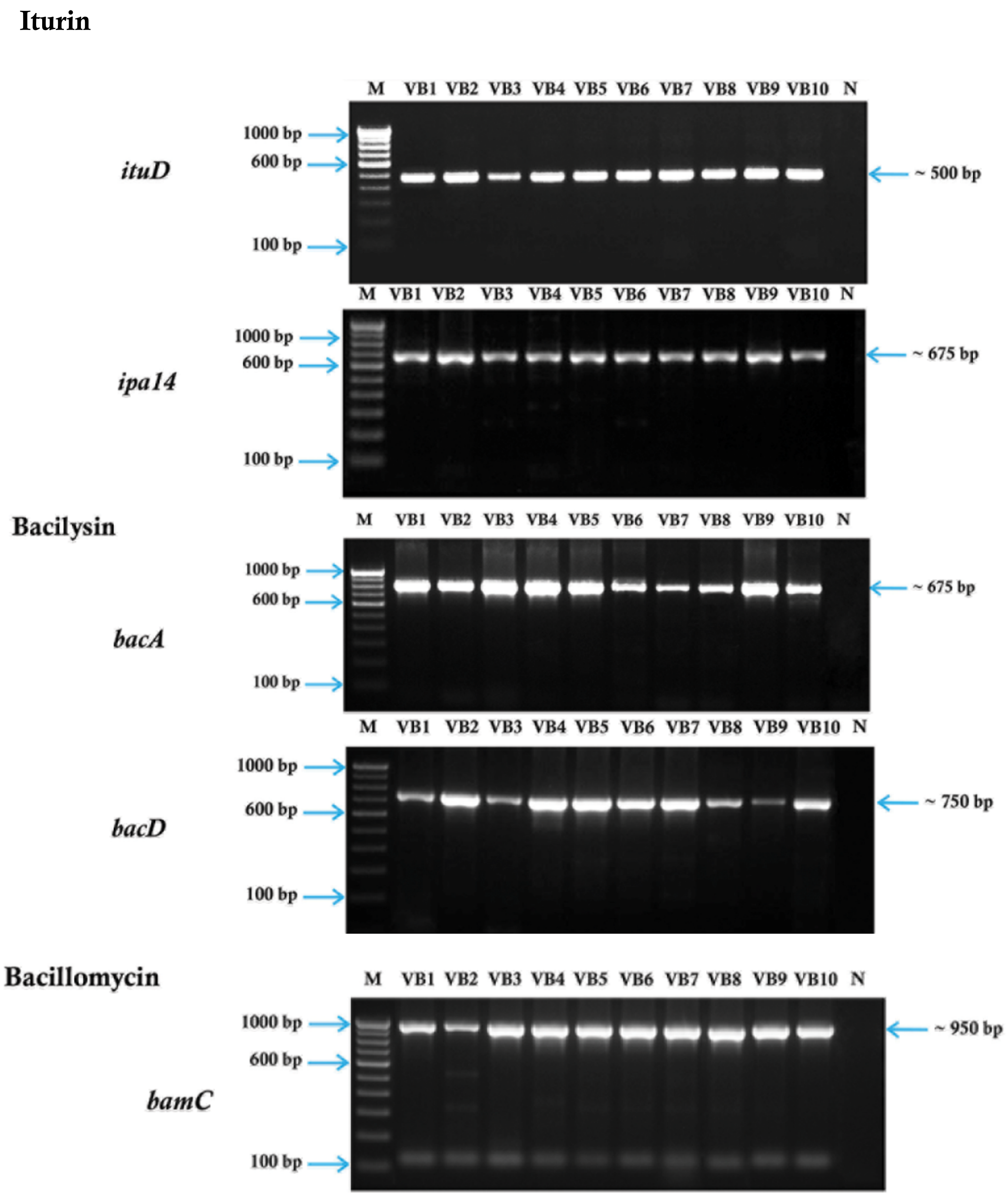

Supplement: Supplementary file 8 [file Image2.PDF]
