# Supplementary material for: Biocontrol Potentials of Antimicrobial Peptide Producing Bacillus Species: Multifaceted Antagonists for the Management of Stem Rot of Carnation Caused by Sclerotinia sclerotiorum
Source: Front Microbiol. 2017 Mar 24;8:446. doi: 10.3389/fmicb.2017.00446 (PMC5364326; doi:10.3389/fmicb.2017.00446)

Figure S4. ***In vitro*** **efficacy of** ***Bacillus*** **species in the suppression of sclerotial production through the secretion of antifungal volatiles assessed through partition plate technique**

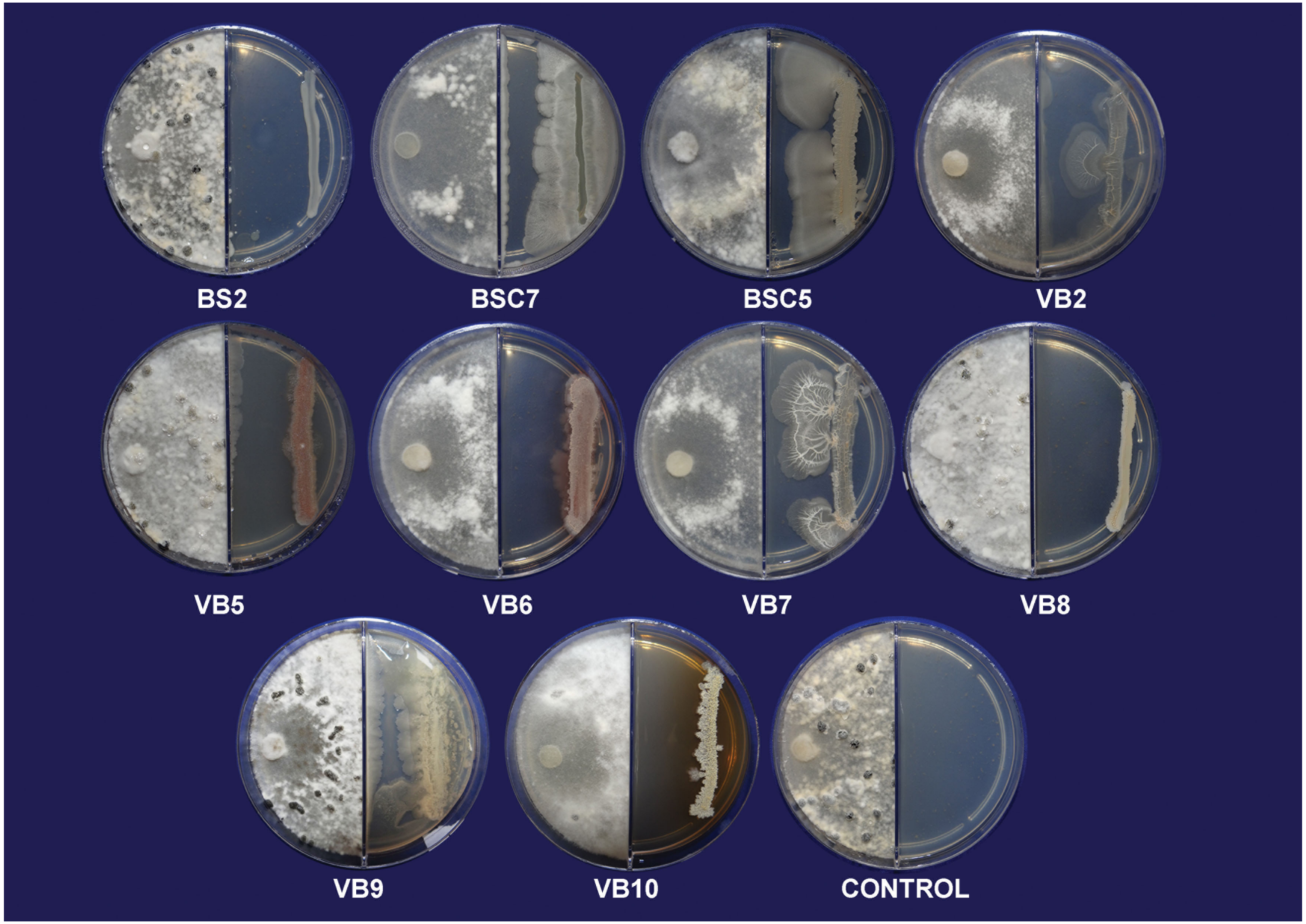

Supplement: Supplementary file 10 [file Image4.PDF]
