# Supplementary material for: Biocontrol Potentials of Antimicrobial Peptide Producing Bacillus Species: Multifaceted Antagonists for the Management of Stem Rot of Carnation Caused by Sclerotinia sclerotiorum
Source: Front Microbiol. 2017 Mar 24;8:446. doi: 10.3389/fmicb.2017.00446 (PMC5364326; doi:10.3389/fmicb.2017.00446)

Figure S5. Antifungal activity of crude metabolites of *B. amyloliquefaciens* (VB2 & VB7) against mycelial growth of *S. sclerotiorum*

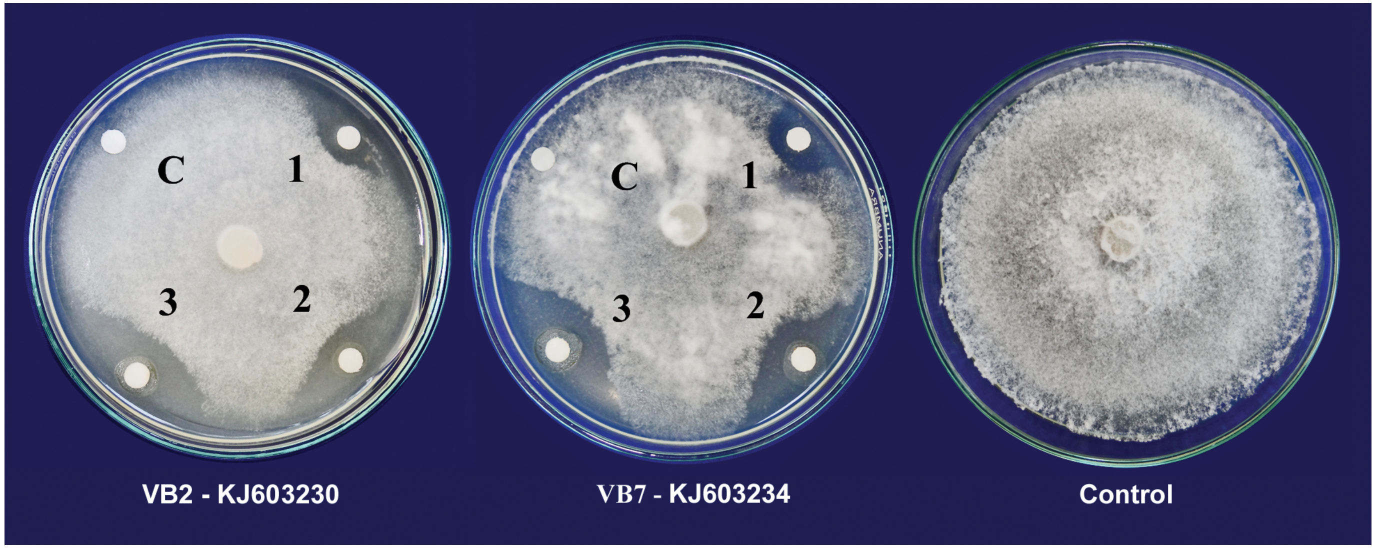

Supplement: Supplementary file 11 [file Image5.PDF]
